# Cooperation between HIV-1 integrase natural polymorphism K156N and 3′PPT mutations in dolutegravir monotherapy failure

**DOI:** 10.1093/jac/dkag033

**Published:** 2026-02-04

**Authors:** Jolieke A T van Osch, Jolanda J C Voermans, Haajar Ouzerne, Alicia B H Cromme, Ehikioya Azugbene, Mike Voskamp, Zoë Krullaars, Rizwan Mahmud, Ronald J Overmars, Alicja U Gorska, Cynthia Lungu, David A M C van De Vijver, Rob A Gruters, Jeroen J A van Kampen, Thibault Mesplède

**Affiliations:** Viroscience Department, Erasmus University Medical Center, Rotterdam, The Netherlands; Viroscience Department, Erasmus University Medical Center, Rotterdam, The Netherlands; Viroscience Department, Erasmus University Medical Center, Rotterdam, The Netherlands; Viroscience Department, Erasmus University Medical Center, Rotterdam, The Netherlands; Viroscience Department, Erasmus University Medical Center, Rotterdam, The Netherlands; Viroscience Department, Erasmus University Medical Center, Rotterdam, The Netherlands; Viroscience Department, Erasmus University Medical Center, Rotterdam, The Netherlands; Viroscience Department, Erasmus University Medical Center, Rotterdam, The Netherlands; Viroscience Department, Erasmus University Medical Center, Rotterdam, The Netherlands; Viroscience Department, Erasmus University Medical Center, Rotterdam, The Netherlands; Viroscience Department, Erasmus University Medical Center, Rotterdam, The Netherlands; Department of Pathology, Erasmus University Medical Center, Rotterdam, The Netherlands; Viroscience Department, Erasmus University Medical Center, Rotterdam, The Netherlands; Viroscience Department, Erasmus University Medical Center, Rotterdam, The Netherlands; Viroscience Department, Erasmus University Medical Center, Rotterdam, The Netherlands; Viroscience Department, Erasmus University Medical Center, Rotterdam, The Netherlands

## Abstract

**Objectives:**

Mutations in the 3′-polypurine tract (3′PPT) of HIV-1 have been observed under pressure with two integrase strand transfer inhibitors, dolutegravir and cabotegravir. In the DOMONO randomized clinical trial, 3′PPT mutations emerged in a participant who experienced treatment failure under dolutegravir monotherapy. To understand the basis for this rare mutational pathway, we examined baseline viral sequences and identified the K156N natural polymorphism. Given the role of K156 in viral DNA binding, the potential relationship between K156N and 3′PPT mutations was further investigated.

**Methods:**

We assessed the impact of K156N on integrase using *in silico* modelling and biochemical assays with recombinant proteins. Infectivity, replicative capacity, and drug susceptibility of viruses carrying K156N, 3′PPT mutations, or both were measured. Viral evolution was assessed in cell culture.

**Results:**

Structural models indicated that K156N altered viral DNA binding. K156N reduced strand transfer activity through decreased affinity for the LTR but increased 3′-processing. The K156N virus had normal infectivity, whereas the 3′PPT mutations decreased infectiousness sixfold and lowered maximal infectivity. K156N partially compensated for this defect, but maximal infectivity remained diminished. K156N also partially compensated for defects in replicative capacity imposed by 3′PPT mutations. K156N alone did not confer resistance against dolutegravir, nor did it increase the modest (2.5-fold) resistance conferred by the 3′PPT mutations. K156N alone promoted the spontaneous emergence of 3′PPT mutations distinct from those seen in DOMONO.

**Conclusions:**

These findings establish a direct functional relationship between natural variation in HIV-1 integrase and the emergence of 3′PPT mutations. People harbouring a virus with the K156N natural polymorphism may be predisposed to developing 3′PPT mutations upon failure with DTG. However, the clinical relevance of this association remains to be established.

## Introduction

Dolutegravir has become the cornerstone of HIV treatment worldwide due to its high potency, tolerability, barrier to resistance, and open patent.^1^ As its use has expanded to low- and middle-income countries, concerns have emerged about the potential for resistance development against this drug and its transmission at the population level.^2^ In addition, its high barrier to resistance has prompted investigations into noncanonical pathways of resistance outside the integrase coding region. One such potential mechanism involves the 3′ polypurine tract (3′PPT).^3,4^ The clinical relevance of 3′PPT mutations remains unclear.

The 3′PPT is a short RNA sequence located immediately upstream of the 3′-long terminal repeat (LTR) at the end of the HIV-1 genome, overlapping the *nef* gene. It is mono-directionally resistant against degradation by the RNaseH activity of the reverse transcriptase and serves as a primer for the initiation of plus-strand DNA synthesis.

Importantly, because the 3′PPT is located immediately upstream of the 3′-LTR in the RNA genome, it is essential for generating a conserved 5′-ACTG-3′ sequence in 5′ of the double-stranded reverse-transcribed HIV-1 DNA genome. HIV-1 integrase specifically binds the LTR ends to catalyse integration into the host genome.^5^ Integration starts with 3′-processing, which removes the conserved 5′-GT-3′ dinucleotides from the 3′ ends of the viral dsDNA. This removal exposes 3′-hydroxyl groups at both DNA ends, which mediate a nucleophilic attack on the host DNA during a second step, called strand-transfer. Integrase strand-transfer inhibitors (INSTIs), the drug class that includes dolutegravir, potently block strand-transfer by binding the integrase-viral DNA complex.^6–8^ Cell-free studies showed that mutations in the LTR termini can impair integrase function and reduce INSTI binding.^9^

The first direct evidence that HIV-1 can acquire resistance against dolutegravir via 3′PPT mutations came from long-term cell-based selection experiments conducted under high dolutegravir concentrations.^10^ These experiments led to the emergence of mutations in the coding region of nef, some of which were in the 3′PPT region, notably within the highly conserved guanine-hexamer (G-stretch) that directly borders the end of the 3′-LTR.^10^ It has been suggested that co-infection with HTLV-1 of the cells used may have facilitated the selection of these mutations.^11^ Although these variants conferred high-level resistance against dolutegravir and two other INSTIs, raltegravir and elvitegravir, they also severely impaired viral replication,^12^ in some cases to the extent that phenotypic characterization was not feasible.^13^ One proposed resistance mechanism involved the extension of the 5′LTR during reverse transcription, although this remains unconfirmed.^5,14^ Subsequent studies showed that alternative 3′PPT mutations can also confer high-level resistance to dolutegravir by enabling HIV-1 replication without integration, through the accumulation of unintegrated episomal DNA forms.^11,15,16^

In clinical settings, the 3′PPT is highly conserved, particularly the G-stretch.^17–19^ Some of the other nucleotides in the 3′PPT are naturally polymorphic and have been reported to change over time, but no association with dolutegravir resistance has been established.^17–20^ To date, the only clinical case linking 3′PPT mutations directly with treatment failure on dolutegravir was reported in the DOMONO randomized clinical trial, in a participant assigned to dolutegravir monotherapy.^4^ In this participant (DOMONO-10), mutations emerged in the G-stretch of the 3′PPT (5′-GGGAGC-3′) at suspected virological failure (SVF), when the plasma viral load was 313 copies of RNA/mL.^4^ Notably, the mutations were observed in viral RNA, consistent with active replication.

Later studies have suggested that these specific G-stretch mutations do not confer measurable resistance against dolutegravir in cell-based assays (FC = 1.3 without statistical significance).^13,21,22^ Two studies reported no impact on viral infectivity, using replication-competent vectors.^13,21^ In contrast, a third study reported a 95% reduction in infectivity, but used a non-replicative vector system, which may have affected replication dynamics.^22^ None of these studies found an effect of the G-stretch mutations on susceptibility to non-nucleoside reverse transcriptase inhibitors (NNRTIs).^13,22^

To further understand the factors underlying the uniqueness of the DOMONO-10 case, we re-examined integrase sequencing data and observed the K156N natural polymorphism, present both at baseline and at virological failure.^12^ Given the established role of K156 in viral DNA binding and integrase function,^23–26^ we reflected that this natural polymorphism may have been involved in facilitating the development of 3′PPT G-stretch mutations in this person. Notably, K156N has also been involved in changes in susceptibility to multiple INSTIs.^25,27,28^ Here, we report the functional characterization of K156N in the context of the 3′PPT G-stretch mutations observed during dolutegravir monotherapy failure in the DOMONO-10 case.

## Methods

### Cells and reagents

TZM-bl reporter cells (#ARP-8129) were obtained from the NIH AIDS Reagent program, Division of AIDS, NIAID, NIH, via BEI Resources. HEK293T cells and TZM-bl cells were cultured at 37°C with 5% CO_2_ in DMEM supplemented with 100 U/mL of penicillin, 100 μg/mL of streptomycin, and 10% foetal bovine serum. Dolutegravir (1051375-19-9) was purchased from MedChem Express. Islatravir (865363-93-5) was purchased from BioConnect Life Sciences.

### Plasmids

The pNL4-3 infectious molecular clone (#ARP-114), pNL4.3 ΔEnv eGFP reporter plasmid (#ARP-11100), and the pET15b-integrase (IN) plasmid (#ARP-2958) were obtained from the NIH AIDS Reagent program.^29,30^ The K156N natural polymorphism was added to the pNL4.3, pNL4.3 ΔEnv eGFP, and pET15b-IN plasmids by site-directed mutagenesis, as previously published.^31^ Primers for mutagenesis were as follows: K156N-sense 5′-TTACCTGTCCTATAATTTTCTTTAATTCATTATTCATAGATTCTATTACTCCTTGAC-3′ and K156N-antisense 5′-GTCAAGGAGTAATAGAATCTATGAATAATGAATTAAAGAAAATTATAGGACAGGTAA-3′. The 3′PPT G-stretch mutations found in DOMONO-10 were added to WT and K156N pNL4.3 and pNL4.3 ΔEnv eGFP plasmids by site-directed mutagenesis with the 3PPT-sense 5′- GAGTGAATTAGCCCTTCCAGTGCTCCCTTTTCTTTTAAAAAGTGG-3′ and 3PPT-antisense 5′- CCACTTTTTAAAAGAAAAGGGAGCACTGGAAGGGCTAATTCACTC-3′ primers. Successful mutagenesis was confirmed by sequencing.

### *In silico* structural modelling

Structural modelling of HIV-1 integrase was performed as previously published.^31,32^ Initial analyses were performed using the 6PUT, 9C9M and 8W09 published structures.^33–35^  *In silico* mutagenesis and structure refinement were performed and visualized with PyMol Graphics System v2.3.4 (Schrodinger LLC), Maestro v13.2.128 (Schrodinger LLC), and ModRefiner.^31,32,36,37^ To model integrase complexes bound to unprocessed and 3′-processed LTR, *de novo* structural models were created using AlphaFold3.^38^

### Integrase protein purification

Recombinant HIV-1 integrase proteins were purified as published before.^31,37^ Eluted proteins were quantified by absorbance, aliquoted, and stored at −80°C.

### Strand transfer assays

The strand transfer activity of recombinant integrase proteins was measured using the protocol described in Xiao *et al.,*^31^ with the notable difference that target DNA was labelled with the FAM fluorophore in 5′ and the BHQ1 quencher in 3′ (Table 1). The strand transfer assay involved a pre-processed dsDNA LTR mimetic: sense (5′-ACCCTTTTAGTCAGTGTGGAAAATCTCTAGCA-3′) and antisense (5′-ACTGCTAGAGATTTTCCACACTGACTAAAAG-3′). When indicated, an extended antisense LTR mimetic (5′-**C**ACTGCTAGAGATTTTCCACACTGACTAAAAG-3′) was used to mimic a potential extension of the LTR caused by the mutation observed in DOMONO-10.^4^ For the delta-protein tests, assays were performed in the presence of 400, 800, and 1600 nM recombinant integrase and 20 nM of target and LTR DNA. For delta-target DNA assays, 400 nM integrase and 100 nM of LTR DNA were incubated with serial dilutions of target DNA from 250 nM to 4.3 pM. For delta-LTR assays, 400 nM integrase and 100 nM target DNA were incubated in the presence of serial dilutions of LTR DNA (250 nM to 4.3 pM). Enzymatic parameters were derived using GraphPad Prism v. 10.4.2 (GraphPad Prism) from at least three separate experiments each performed in duplicate, with two different batches of purified proteins, as previously published.^31,37^

**Table 1.** List of oligonucleotides, probes, and DNA duplexes used in the strand-transfer and 3′-processing assays

Illustrations				
Type	Name		Sequence	
Oligonucleotide	Target DNA sense	5'	TGACCAAGGGCTAATTCACT	3'
Oligonucleotide	Target DNA antisense	5'-6FAM	6FAM-AGTGAATTAGCCCTTGGTCA-BHQ1	3'-BHQ1
Oligonucleotide	LTR sense	5'	ACCCTTTTAGTCAGTGTGGAAAATCTCTAGCA	3'
Oligonucleotide	LTR antisense	5'	ACTGCTAGAGATTTTCCACACTGACTAAAAG	3'
Oligonucleotide	LTR antisense DOMONO-10	5'	**C** ACTGCTAGAGATTTTCCACACTGACTAAAAG	3'
Oligonucleotide	LTR probe sense	5'	ACTGGAAGGGCTAATTTGGTTGTGGAAAATCTCTAGCAGT	3'-BHQ1
Oligonucleotide	LTR probe antisense	5'-HEX	ACTGCTAGAGATTTTCCACAACCAAATTAGCCCTTCCAGT	3'
Duplex	Target DNA	5'	TGACCAAGGGCTAATTCACT	3'
			||||||||||||||||||||	
		3'-BHQ1	ACTGGTTCCCGATTAAGTGA	5'-6FAM
Duplex	LTR	5'	ACCCTTTTAGTCAGTGTGGAAAATCTCTAGCA	3'
			|||||||||||||||||||||||||||||	
		3'	—--GAAAATCAGTCACACCTTTTAGAGATCGTCA	5'
Duplex	LTR_DOMONO-10	5'	ACCCTTTTAGTCAGTGTGGAAAATCTCTAGCA	3'
			|||||||||||||||||||||||||||||	
		3'	—--GAAAATCAGTCACACCTTTTAGAGATCGTCA**C**	5'
Duplex	LTR probe	5'	ACTGGAAGGGCTAATTTGGTTGTGGAAAATCTCTAGCAGT	3'-BHQ1
			||||||||||||||||||||||||||||||||||||||||	
		3'	TGACCTTCCCGATTAAACCAACACCTTTTAGAGATCGTCA	5'-HEX

The extra cytidine at the 5′-end of the 3PPT-LTR is underlined and bolden. Abbreviations: 6-FAM (6-carboxyfluorescein), Hex (hexachloro-fluorescein), and BHQ-1 (black hole quencher-1).

### Dolutegravir inhibition of strand-transfer in cell-free enzymatic assays

We performed a strand-transfer assay in the presence of serially diluted dolutegravir, using concentrations ranging from 20 to 0.16 nM. Global nonlinear regression was performed on the progress curves, and a mixed model of inhibition was applied without constraining the Vmax values since dolutegravir inhibited the plateauing of this reaction. Apparent Km values were fitted based on the results of the above-described experiments performed in the absence of dolutegravir (1.8 nM for WT and 4.1 nM for K156N). From this analysis, inhibitory constants (Ki values) were derived for the WT and K156N recombinant proteins.

### 3′-processing assays

The 3′-processing activity of recombinant integrase proteins was measured using a protocol derived from Han Y *et al.,* and He HQ *et al*.^39,40^ The oligonucleotides used are shown in Table 1. One strand was labelled with a HEX fluorophore in 5′, and the complementary strand carried a black hole quencher 1 (BHQ-1) at the 3′ end. In the annealed duplex, fluorescence emission was quenched. Upon 3′-processing by integrase, the quencher was removed from the duplexes, resulting in time-dependent fluorescence production. A duplex substrate was prepared by heat denaturation followed by slow annealing, yielding >80% duplex formation. Reactions were performed at 37°C for 2h in a buffer containing 25 mM morpholino-propanesulfonic acid (MOPS, pH = 6.8), 95 mM NaCl, 100 µg/mL BSA, 2 mM MgCl_2_, and 2 mM MnCl_2_. Purified recombinant proteins (400 nM) were incubated with serial dilutions of LTR probe duplexes at concentrations ranging from 10 μM to 20 nM. Initial reaction velocities (V_0_) were measured by quantifying fluorescence over time and plotted against LTR concentrations using GraphPad Prism v 10.6.1 (GraphPad Software, LLC). The data best fitted an allosteric sigmoidal model, compared to the Michaelis-Menten function (*P* < 0.002), consistent with reported integrase kinetics, and V_max_ and K_half_ parameters were derived accordingly. Five separate experiments were performed with separate batches of proteins.

### Virus production

The pNL4.3 and pNL4.3 ΔENV eGFP WT, K156N, 3′PPTmut, and K156N + 3′PPTmut plasmids were produced by transfection into 293T cells as published previously.^31^ Viral stocks were quantified by RT-qPCR as published previously.^41^

### Resistance and infectivity assays

Both assays were performed using TZM-bl reporter cells, as published previously.^31^ Specifically, 30 000 TZM-bl cells were seeded per well in 96-well plates on day 0. Twenty-four hours later, serial dilutions of ΔENV virus stocks were added to the cells and incubated at 37°C for 1h, after which the inoculum was removed and replaced with fresh medium. Forty-eight hours post-infection, cells were lysed with 60 μL of Reporter Lysis Buffer (Promega). Thirty microliters of the lysate were mixed with 30 μL of Luciferase Assay System (Promega, E4030), incubated for 10 minutes at room temperature, and luminescence was measured on a Tecan Infinite 200 PRO plate reader (Tecan). Data from at least three experiments with two distinct viral batches were normalized to the WT virus, combined, and analysed using GraphPad Prism v.10.4.2 (GraphPad). Half-infectious doses (ID_50_) and maximal infectivity were derived using the same software by nonlinear regression.

For resistance assays, TZM-bl reporter cells were plated as described above. On day 1, serial dilutions of dolutegravir ranging from 11 μM to 0.01 nM were added to the cells and incubated for 1h at 37°C before infection. The medium was then removed, and cells were infected with normalized amounts of ΔENV viruses. After another hour of infection, the inoculum was removed and replaced with fresh medium. Forty-eight hours later, luciferase activity was measured as described above. At least 3 experiments with two distinct viral batches were combined for analysis. Normalized data were plotted, and half-inhibitory concentrations (IC_50_) and maximal inhibition were derived using GraphPad Prism v.10.4.2 (GraphPad).

### Replicative capacity

In contrast to infectivity and resistance assays, which were performed as single-cycle assays with ΔENV viruses, replicative capacity was assessed using fully replication-competent viruses. TZM-bl cells were infected with normalized amounts of WT, K156N, 3′PPT, and K156N + 3′PPT viruses for 24, 48 and 96 hours post-infection, and luciferase production was measured as described above. Results from four independent experiments were analysed using GraphPad Prism v.10.4.2 (GraphPad). Areas under the curves (AUC) were calculated for each virus and normalized to the WT virus, arbitrarily set at 100%. Lower AUC values indicate lower replicative capacity.

### Cell-based selection experiments

WT and K156N viruses were cultured for 2 weeks with peripheral blood mononuclear cells (PBMCs), following a protocol adapted from a previous publication.^42^ PBMCs were isolated from commercially-obtained buffy coats from three healthy donors (Sanquin, NL). At day 0, PBMCs were thawed, pooled, and activated in RPMI1640 supplemented with 100 U/mL of penicillin, 100 μg/mL of streptomycin, 10 μg/mL of phytohemagglutinin A (PHA), 20 U/mL of human interleukin-2 (IL-2), and 10% decomplemented foetal bovine serum. Three days later, 20 million activated PBMCs were infected with the WT or K156N viral stock normalized at 5×10^5^ RNA copies. At the end of week 2, viral RNA was purified using the QiaAmp viral RNA kit (Qiagen), and integrase and 3′PPT were sequenced using Sanger sequencing. Specifically, the 3′PPT region was reverse transcribed and amplified using the following primers: 5′-AGAGTTAGGCAGGGATATTCACC-3′ and 5′-GCACTCAAGGCAAGCTTTATTGAGGCT-3′ and the SuperScript^™^ III One-Step RT-PCR System with Platinum^™^ Taq DNA Polymerase (ThermoFisher Scientific). Amplicons were PCR-purified using the QIAquick PCR Purification Kit for PCR Cleanup (Qiagen), and nested amplification was performed using the following primers: 5′-CCAGCAGCAGATGGGGTGGGAGCAG-3′ and 5′-GCTTATATGCAGCATCTGAGGG-3′ and the PfuUltra II Fusion high-fidelity DNA polymerase (Agilent). The same nested primers were used for Sanger sequencing on a Hitachi 3500xL Genetic Analyzer (Applied Biosystems). In addition, nested amplicons were analysed by next-generation sequencing using the Oxford Nanopore Technology via the commercial platform Plasmidsaurus (https://plasmidsaurus.com/). Raw reads were trimmed for quality, aligned to the HIV-1 reference sequence AF324493.2, and mutation frequencies were quantified using Geneious Prime 2026.0.2 (GraphPad Software LLC).

## Results

### The K156N natural polymorphism affects LTR DNA binding *in silico*

**Figure 1.**
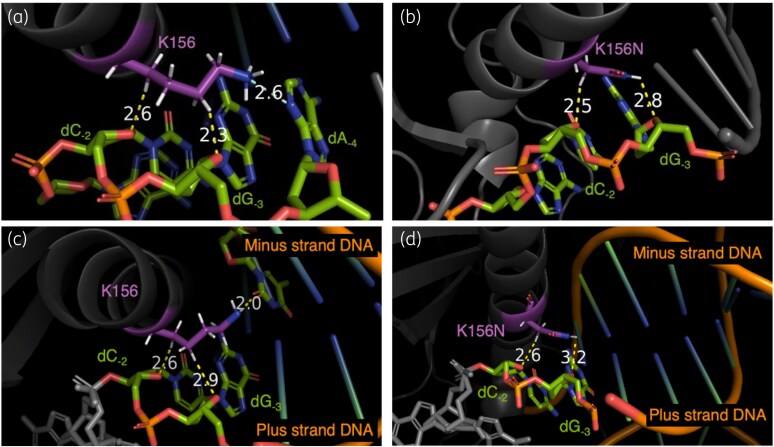
Structural impact of the K156N natural polymorphism on HIV-1 integrase interaction with viral DNA. (a–b) Structural models of wild-type (a) and K156N (b) integrase bound to unprocessed viral DNA were generated *de novo* using AlphaFold3. (c–d) Structural models of WT (c) and K156N (d) were adapted from the cryo-EM structure PDB ID:8W09, representing integrase bound to processed viral DNA. In all panels, K156 and K156N are shown in the middle (in purple), functional interactions are indicated with dotted lines and labelled with corresponding distances in Ångström. DNA is shown at the bottom and at the top of the panels (in teal) with functional residues labeled accordingly. Non-interacting chains and residues are shaded or hidden for clarity.

Although the involvement of K156 in viral DNA binding is well established,^7,25^ no crystal structure of the K156N HIV-1 integrase protein bound to the LTR is available. To address this limitation, we performed *in silico* structural modelling using wild-type and K156N integrase proteins in complex with unprocessed or 3′-processed LTR DNA, with or without dolutegravir. In models with unprocessed LTR DNA and no drug, K156 is positioned in the minor groove between the two DNA strands and forms two strong hydrogen bonds with the deoxyribose sugar of the nucleotides at positions −2 and −3 (Figure 1a). Additionally, a stabilizing polar interaction was observed between the ε-amino group of K156 and the imidazole ring of the adenine base at position −4. This interaction was also evident in the experimentally determined structure 6PUT.^35^ When K156N was introduced into the same DNA-bound complex, the two primary hydrogen bonds at positions −2 and −3 were preserved (Figure 1b). However, the shorter side chain of asparagine failed to reach the adenine at position −4, eliminating the third stabilizing interaction in all models. A similar pattern was observed in models with 3′-processed LTR DNA where K156 formed strong hydrogen bonds at −2 and −3 and a polar contact with the −4 adenine, which was lost in the K156N mutant (not shown). In a slightly lower resolution cryo-electron microscopic structure, another form of interaction between K156 and the LTR DNA was observed.^33^ In this case, the ε-amino group of K156 forms a directional hydrogen bond with the O2 atom of thymine at position −4 on the minus-strand DNA, opposite to the 3′-processed strand (Figure 1c). The donor hydrogen was positioned within 2.0 Å, consistent with a strong hydrogen bond, which was lost in K156N models (Figure 1c-d). Overall, K156N preserved essential DNA interactions but eliminated a third potentially stabilizing interaction. These findings suggest that K156N may subtly reduce the LTR DNA-binding affinity of integrase, which could have functional consequences in the selection of 3′PPT mutations.

### K156N modulates integrase strand-transfer activity by decreasing LTR DNA binding

**Figure 2.**
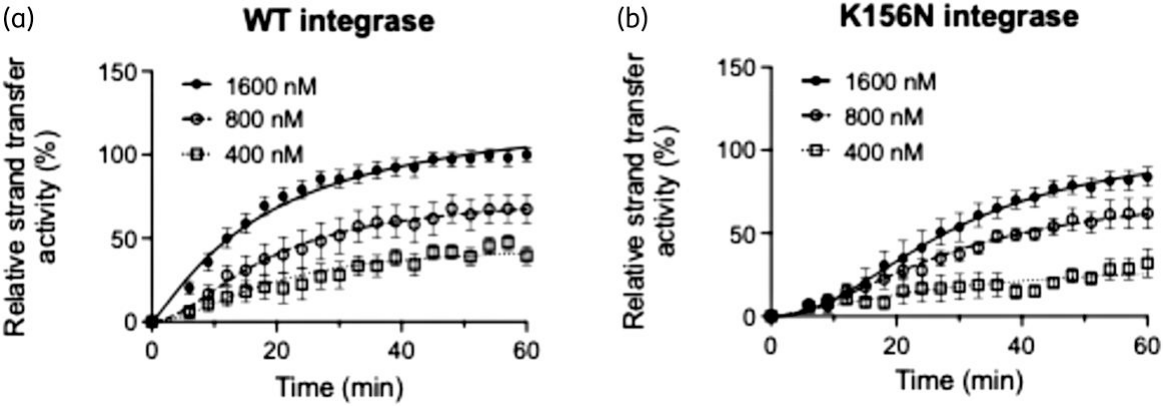
Strand transfer activity of WT and K156N HIV-1 integrase proteins. (a–b) Strand transfer assays were performed with increasing concentrations of recombinant WT (a) or K156N (b) HIV-1 integrase proteins. Results represent the mean of five independent experiments. Signals were normalized to the maximal value obtained with 1600 nM WT enzyme, set arbitrarily at 100%. Error bars indicate standard deviation (SD).

Given the impact of K156N on LTR binding and its known function in DNA interactions, we next measured the strand-transfer activity of integrase proteins with and without K156N. K156N was less effective at strand-transfer, yielding lower signals than the WT enzymes at all protein concentrations (Figure 2). To further investigate the underlying cause of this defect, we performed strand-transfer assays with various concentrations of substrates, i.e. LTR and target DNA. Our results show no difference in target DNA binding affinity for WT versus K156N (Table 2). In contrast, K156N showed a twofold decrease in LTR binding, which is comparable to that observed with R263K, G118R, and S153F/Y.^31,37,43^ We also performed assays in the presence of dolutegravir that showed no difference in the *Ki* values for the WT and K156N proteins (Table 2). Our Ki data were compatible with previously published reports.^44–48^

**Table 2.** Strand transfer assay parameters of WT and K156N HIV-1 integrase proteins

Protein	Apparent K_m_ (target DNA)	Apparent K_m_ (WT LTR DNA)	Apparent K_m_ (3PPT-LTR DNA)	DTG K_i_
WT	3.2 [1.7–4.8]	1.8 [1.3–2.3]	39 [31–49]	0.9 [0.7–1.0]
K156N	3.2 [2.1–4.4]	4.1 [3.5–5.4]^a^	33 [25–43]	0.8 [0.8–0.9]

Apparent Km values reflect the affinity of integrase for the LTR or target DNA substrate. A modified LTR (3PPT-LTR), containing an additional cytidine at the 5′ end, was used to mimic the 5′-LTR extension potentially resulting from the DOMONO-10 3′PPT mutations. Kᵢ values represent the inhibitory constant of dolutegravir (DTG) for each integrase variant. Data were derived from strand transfer assays conducted in three independent experiments using two separate protein purification batches.

^a^Statistically significant differences from WT are indicated (Student *t*-test, *P* < 0.05).

### K156N does not improve strand-transfer with a 5′-extended LTR

Next, we wanted to evaluate whether the K156N polymorphism could facilitate strand-transfer with an extended viral DNA end (3PPT-LTR) that mimics the potential elongation of the LTR caused by the 3′PPT G-to-C mutation found immediately in 5′ of the LTR in DOMONO-10 (Table 1). When this extended DNA substrate was used, strand transfer activity was reduced by approximately 20-fold relative to the WT LTR. Importantly, both WT and K156N integrase proteins exhibited similarly reduced activity, indicating that K156N does not confer any catalytic disadvantage for integration when the viral DNA end is extended (Table 2).

### K156N increases 3′-processing

To determine the effect of K156N on the first step of HIV-1 integration, we performed 3′-processing assays as previously described, with minor modifications.^39,49^ Compared to the WT integrase, the K156N protein exhibited a significant 1.4-fold increase in V_max_ for 3′-processing (Table 3), despite a marked (13-fold) increase in K_half_, indicative of reduced apparent binding affinity for the LTR substrate.

**Table 3.** 3′-processing kinetic parameters of WT and K156N HIV-1 integrase proteins

Protein	Khalf	FC Khalf	Vmax	FC Vmax
WT	0.3 [0.2–0.5]	—	14 341 [13 217–15 966]	—
K156N	4 [1.9–12.5]^a^	13	19 716 [16 248–26 410]^a^	1.4

3′-processing activity was analysed by fitting the data to an allosteric sigmoidal model. Apparent Khalf values represent the LTR substrate concentration required to achieve half-maximal activity and were used here to measure integrase–LTR affinity. Vmax denotes the maximal catalytic velocity. Parameters were derived from five independent 3′-processing experiments and performed using at least two separate batches of purified integrase proteins.

^a^Statistically significant differences relative to WT are indicated (Student's *t*-test, *P* < 0.05).

### K156N counteracts infectivity defects without increasing dolutegravir resistance

Biochemical data indicated that the K156N improved 3′-processing activity but reduced strand-transfer efficiency, possibly resulting in a neutral overall effect on integration, and showed no effect on dolutegravir susceptibility. To further investigate the functional interaction between K156N and the 3′PPT mutations identified in DOMONO-10, we examined their combined effects on infectivity and dolutegravir susceptibility in cell culture.

K156N alone had no effect on infectivity (Table 4). In contrast, the 3′PPT mutations increased the half-infectious dose (ID₅₀) approximately sixfold relative to the WT virus and lowered the maximal infectivity by 13%. Notably, the K156N + 3′PPT double mutant restored ID₅₀ values to WT levels, suggesting that K156N compensated for the infectivity defect caused by the 3′PPT mutations. However, this compensation was partial as, although the ID₅₀ was restored, the maximal infectivity of the double mutant plateaued at lower levels (63%) than the 3′PPT mutant (87%). Similar results were obtained when fully replication-competent viruses were used to assess replicative capacity (Table 4). Replicative capacity of WT and K156N viruses was not significantly different. In contrast, the 3′PPT mutations reduced replicative capacity to approximately 25% of WT levels, whereas viruses carrying both K156N and the 3′PPT mutations exhibited ∼80% replicative capacity, indicating partial compensation. Regarding drug susceptibility, K156N had no effect when tested individually (Table 4). The 3′PPT mutations alone conferred low-level resistance to dolutegravir. Contrary to our expectations, the combination of K156N with 3′PPT mutations did not increase dolutegravir resistance relative to the 3′PPT mutations alone; instead, it rendered the difference from wild-type (WT) virus no longer statistically significant. None of the mutations or combinations altered susceptibility to the nucleoside reverse transcriptase translocation inhibitor (NRTTI) islatravir, used as a control. Similar to a previous report, we observed increases in the plateau phase of the dose-response curves with dolutegravir when the 3′PPT and K156N + 3′PPT mutant viruses were used, consistent with incomplete suppression at high drug concentrations.^21^ This was not observed with islatravir.

**Table 4.** Infectivity, replicative capacity, and antiretroviral susceptibility of HIV-1 subtype B viruses

	Infectivity		Relative replicative capacity	Susceptibility			
Genotype	ID_50_	Maximal infectivity		FC (DTG)	Maximal inhibition (DTG)	FC (ISL)	Maximal inhibition (ISL)
WT	1 [0.9–1.1]	100%	100%	1 [0.7–1.5]	91%	1 [0.9–1.12]	99%
K156N	0.9 [0.9–1]	99%	134%	1.1 [0.8–1.6]	90%	1.1 [0.9–1.4]	97%
3'PPT	6.6 [6.5–6.7]^a^	87%^a^	22%^a^	2.5 [1.8–3.4]^a^	69%^a^	0.9 [0.8–1]	98%
K156N + 3'PPT	0.9 [0.9–0.9]	63%^a^	83%^a^	1.6 [1.2–2.2]	68%^a^	1.1 [0.9–1.3]	96%

DTG: dolutegravir; ID50: half-infectious dose; ISL: islatravir

Infectivity is reported as the half-infectious dose (ID₅₀), expressed relative to wild-type (WT) with 95% confidence intervals, and as maximal infectivity (% of WT). Replicative capacity is expressed relative to that of the WT virus, arbitrarily set at 100%. Antiretroviral susceptibility to dolutegravir (DTG) and islatravir (ISL) is shown as fold-change (FC) relative to WT with 95% confidence intervals, and as maximal per cent inhibition. Values represent the compilation of three independently performed experiments.

^a^statistically different from the WT (Student *t*-test, *P* < 0.05)

### K156N promotes spontaneous selection of 3′PPT mutations in the absence of drug pressure

Given the apparent ability of K156N to partially compensate for the fitness defects associated with 3′PPT G-stretch mutations, we reflected that drug resistance selection with this natural polymorphism might facilitate the emergence of 3′PPT mutations under dolutegravir selective pressure in cell culture. The resistance selection experiments using the WT or K156N virus under dolutegravir drug pressure failed to yield resistance mutations in either the integrase coding region or the 3′PPT. However, we unexpectedly observed the rapid and spontaneous emergence of a mixture of mutations in the 3′PPT after 2 weeks of culture in the absence of dolutegravir, but only when infections were initiated with K156N (AAAAGAAAA -> AA**R**AGAA**M**A). Notably, the mutations selected were outside the 6-G stretch and distinct from the mutations observed in DOMONO-10. Further examination of sequences encompassing the 3′PPT region by next-generation sequencing (NGS) confirmed the absence of changes in the 600 nucleotides surrounding the 3′PPT. NGS data also confirmed the development of AA**G**AGAA**C**A mutations, represented at 24.7% and 24.3%, respectively, and almost always (98%) occurring in combination. No additional resistance-associated mutations were detected within the duration of these experiments, either in the 3′PPT or in integrase. However, these experiments were limited to a relatively short selection period and therefore did not capture longer-term evolutionary outcomes. Overall, these results indicate that K156N facilitates the selection of mutations in the 3′PPT.

## Discussion

This study shows for the first time a functional relationship between the HIV-1 integrase natural polymorphism K156N and the 3′PPT mutations that were observed during dolutegravir monotherapy failure.

Biochemically, K156N was associated with reduced strand transfer activity due to 2- to 13-fold decreased LTR affinity, depending on the assay employed (Figure 2, Tables 2 and 3). This decrease is consistent with several published reports that have shown K156 to play a critical role in viral DNA binding and strand-transfer,^23,25,50^ as well as our *in silico* modelling (Figure 1) and results from our strand-transfer assays (Figure 2). Importantly, the decrease in DNA binding affinity was specific to the LTR, as binding to target DNA was unaffected by K156N (Table 2).

Somewhat unexpectedly,^25^ K156N enhanced 3′-processing activity in our assays (Table 3). This apparent dissociation between binding and maximal catalytic rate is consistent with a partial uncoupling of substrate recognition from turnover. In this context, the K156N mutation disfavours initial substrate binding while possibly accelerating a downstream, rate-limiting step of the catalytic cycle. Similar trade-offs between affinity and catalytic capacity have been described for other enzymes in which altered conformational dynamics or active-site geometry enhance catalytic flux once a productive enzyme–substrate complex is formed. In this specific case, we believe that this decoupling may be explained by changes in K156N-DNA interactions (Figure 1), which may reduce duplex stabilization and facilitate local strand separation. In turn, this partial destabilization could ease DNA bending or sliding into the catalytic pocket, thereby accelerating 3′-processing or product release. Such a mechanism is consistent with previous structural studies showing that integrase induces conformational strain in the DNA to align the scissile phosphate for cleavage.^35^

The contrasting effects of K156N on strand transfer (twofold decrease in affinity) and 3′-processing (1.4-fold increase in maximal velocity) may be neutral on overall integration efficiency. Consistent with this hypothesis, viruses carrying K156N alone showed normal infectivity and replicative capacity in cell culture (Table 4). This observation is also supported by epidemiological data, as K156N is a naturally occurring polymorphism in 7.4–9% subtype B viruses, which suggests that it does not substantially compromise viral infectivity.^51–53^

In contrast, 3′PPT G-stretch mutations alone led to reduced infectivity, reflected by a ∼6-fold increase in ID₅₀ and decreased maximal infectivity (Table 4) as well as a ∼75% decrease in replicative capacity. Remarkably, our infectivity and replicative capacity data show that K156N partially compensated for the replication defect caused by 3′PPT mutations. The combination of K156N and 3′PPT restored ID₅₀ values to near wild-type levels, but maximal infectivity remained impaired, plateauing at approximately 63%. This phenotype may reflect a shift towards replication via episomal forms, particularly 1-LTR circles, which has been reported for other 3′PPT mutations.^11,15^ In addition, the replicative capacity of fully infectious viruses was also partially restored to ∼80% of the WT levels.

A mechanistic explanation for K156N compensating for 3′PPT mutations comes from our biochemical experiments. When an elongated LTR mimic, designed to reflect the effects of the DOMONO-10 3′PPT mutations, was used in strand transfer assays, activity decreased for both WT and K156N integrase proteins, and the twofold reduction in DNA binding affinity previously observed for K156N with wild-type LTR was no longer present (Table 4). This suggests that K156N may be less functionally impaired when acting on structurally altered LTRs. However, this adaptation may come at the cost of incomplete integration, contributing to the observed infectivity plateau.

A similar plateauing effect was evident in dolutegravir resistance assays (Table 4). Maximal inhibition was lower for both 3′PPT and K156N + 3′PPT viruses than for WT and K156N alone, indicating that a fraction of viral infection occurred even at the highest dolutegravir concentrations. This resistance plateau is consistent with infection via integration-independent episomal forms. Importantly, no such plateau was seen with islatravir.

K156N alone had no measurable impact on dolutegravir susceptibility. The K156N + 3′PPT double mutant did not exhibit greater resistance than the 3′PPT mutations alone. These findings suggest that while K156N may facilitate the emergence or persistence of noncanonical mutations, it does not amplify their resistance phenotype.

Critically, this hypothesis is strongly supported by our cell-based evolution experiments that revealed that K156N, even in the absence of drug pressure, promoted the spontaneous emergence of 3′PPT mutations distinct from those observed in DOMONO-10. These findings align with the clinical observation from the DOMONO trial and suggest a generalizable tendency for viral evolution at the 3′PPT site when K156N is present. However, it is important to note that these experiments were conducted over a limited timeframe, and longer-term serial passage in primary cells may reveal additional evolutionary trajectories or mutational patterns not captured here.^43^ In this context, the absence of resistance mutations in integrase observed in our study is consistent with prior reports indicating that extended selection experiments (>20 weeks) were required for dolutegravir pressure to select integrase mutations.^43,54^

From a clinical perspective, these findings raise important potential considerations. Most importantly, people with viruses harbouring K156N may be more susceptible to the development of 3′PPT mutations upon virological failure with dolutegravir. However, clinical validation of this finding is warranted. The low global prevalence of K156N likely limits its possible impact on public health,^51–53,55^ but it is relatively common in subtype B viruses. Second, other integrase polymorphisms may also act as facilitators of 3′PPT mutations and failure. Given the widespread use of dolutegravir,^2^ both possibilities warrant further clinical investigation.

In conclusion, our findings reveal that the K156N integrase polymorphism creates a permissive environment for the emergence of 3′PPT mutations in cell culture, shedding new light on noncanonical resistance mechanisms to dolutegravir. These data emphasize the interplay between integrase structure, viral DNA 3′-processing, and integration in shaping resistance evolution, and invite further exploration of integrase polymorphisms as modulators of HIV-1 treatment outcomes.
